# A cohort study to investigate sex-specific differences in ANCA-associated glomerulonephritis outcomes

**DOI:** 10.1038/s41598-021-92629-7

**Published:** 2021-06-22

**Authors:** Jennifer Scott, Carolina Canepa, Antonia Buettner, Louise Ryan, Bróna Moloney, Sarah Cormican, Cathal Walsh, Arthur White, Alan D. Salama, Mark A. Little

**Affiliations:** 1 Trinity Health Kidney Centre, Trinity College Dublin, The University of Dublin, Dublin, Ireland; 2 UCL Department of Renal Medicine, Royal Free Hospital, London, UK; 3 Department of Statistics, Trinity College Dublin, The University of Dublin, Dublin, Ireland; 4 Department of Mathematics and Statistics, University of Limerick, Limerick, Ireland; 5 Regenerative Medical Institute (REMEDI) at CÚRAM Centre for Research in Medical Devices, School, College of Medicine, Nursing and Health Sciences, National University of Ireland, Galway, Ireland; 6 Irish Centre for Vascular Biology, Tallaght University Hospital, Tallaght, Dublin, Ireland

**Keywords:** Chronic kidney disease, Glomerular diseases

## Abstract

Data surrounding sex-specific differences in ANCA-associated vasculitis glomerulonephritis (ANCA-GN) outcomes is sparse. We hypothesised that the previously observed increased risk of end-stage kidney disease (ESKD) in males is driven by sex-specific variation in immunosuppression dosing. Patients were recruited to the Irish Rare Kidney Disease Registry or followed by the Royal Free Hospital vasculitis team (2012–2020). Inclusion criteria: prior diagnosis of ANCA-GN (biopsy proven pauci-immune glomerulonephritis) and positive serology for anti-MPO or -PR3 antibodies. Renal and patient survival, stratified by sex and Berden histological class, was analysed. The cumulative- and starting dose/kilogram of induction agents and prednisolone, respectively, was compared between sexes. 332 patients were included. Median follow-up was time 40.2 months (IQR 17.3–69.2). 73 (22%) reached ESKD and 47 (14.2%) died. Overall 1- and 5-year renal survival was 82.2% and 76.7% in males and 87.1% and 82.0% in females, respectively (*p* 0.13). The hazard ratio for ESKD in males versus females, after adjustment for age, ANCA serology, baseline creatinine and histological class was 1.07 (95% CI 0.59–1.93). There was no difference between sexes in the dose/kilogram of any induction agent. We did not observe a strong impact of sex on renal outcome in ANCA-GN. Treatment intensity does not vary by sex.

## Introduction

ANCA-associated vasculitis (AAV) is a rare multi-system autoimmune disease, which results in rapidly progressive glomerulonephritis and immune-mediated destruction of other organs. It is characterised by a necrotising pauci-immune small-vessel vasculitis, with a relapsing and remitting course. AAV comprises three main clinico-pathological syndromes: Microscopic polyangiitis (MPA), Granulomatosis with polyangiitis (GPA) and Eosinophilic granulomatosis with polyangiitis (EGPA). Despite treatment advances, AAV still carries a 2.7-fold increased risk of death compared with the general population^1^ and a significant proportion of patients develop end-stage kidney disease (ESKD)^2–4^. The Berden histological classification system splits patients into four groups according to the predominant histological pattern: focal (≥ 50% normal glomeruli), crescentic (≥ 50% cellular crescents), sclerotic (≥ 50% globally sclerotic glomeruli) and mixed (all others). Multiple studies demonstrate that this classification system is predictive of ESKD risk^5–10^.

Unlike most autoimmune diseases, AAV displays a slight male predominance^11,12^, with the male: female ratio reported as 1.0:0.9^13^. Proteinase 3 (PR3)-ANCA is more prevalent than myeloperoxidase (MPO)-ANCA in males^14^. However, few data exist about sex-specific differences in AAV glomerulonephritis (ANCA-GN) outcomes. A recent study^14^ demonstrated that males have a significantly higher risk of progression to ESKD, particularly in those with crescentic Berden class. A limitation of this study was the lack of personalised treatment data to allow modelling for association with renal outcomes.

We sought to validate the findings of Bjørneklett et al.^14^ using a multi-national cohort, specifically, that the risk of developing ESKD is lower in females, across all histological classes of AAV. We also hypothesised that this difference would be based on a higher effective immunosuppression dose in women, due to lower lean body mass.

## Methods

### Study participants

Participants of this retrospective cohort study were either previously recruited to the national Irish Rare Kidney Disease (RKD) Registry and Biobank^15^, or followed by the vasculitis team at the Royal Free Hospital (RFH), London (United Kingdom (U.K.)), between 2012 and 2020. Participants had to have a prior diagnosis of AAV pauci-immune glomerulonephritis, confirmed on kidney biopsy, with at least three glomeruli^5,14^, and positive serology for anti-MPO or -PR3 antibodies. Patients with secondary vasculitis or dual anti-glomerular basement membrane disease were excluded, in accordance with the European Medicines Agency algorithm^16^. Ethical approval for the study was granted by the Tallaght University Hospital/St. James’s Hospital Joint Research Ethics Committee (ref 2018-10 List 33 (7)). All RKD participants provided written informed consent. In the U.K., the Health Research Authority decision tool determined that additional ethical approval was not required for the RFH cohort, as the study was deemed service evaluation. Our research was performed in accordance with relevant guidelines and regulations.

### Study assessments

We collected data on age, sex, ethnicity, AAV phenotype, ANCA serotype, creatinine (µmol/L), weight (kg) and Berden score on renal biopsy. Treatment data was also collected, including cumulative induction cyclophosphamide and rituximab dose induction and the starting dose of prednisolone. To minimise the risk of incomplete induction treatment data, only cases with a realistic treatment schedule were included: > 3 doses of IV cyclophosphamide or > 1 rituximab dose in the first 6 months of treatment. The observation period was from the date of diagnosis to the occurrence of the first event (ESKD, death or 30th September 2020).

### Statistical analysis

The primary outcome was time to ESKD. ESKD was defined by commencement of renal replacement therapy (and continued for at least 90 days), including dialysis and renal transplantation. The secondary outcome was time to death. A composite of time to ESKD or death was also investigated. The cumulative dose per kilogram of each induction agent was calculated for a subgroup of participants for whom complete induction treatment data was available (n = 189). The starting dose of prednisolone per kilogram was also obtained. Continuous variables are reported as mean (standard deviation, SD) or median (interquartile range, IQR, if not normally distributed), and compared using the independent sample t-test or Mann–Whitney U test, respectively. Categorical variables are summarised by frequency and percentage (%) and compared using the chi-square test. Renal survival was determined using Kaplan–Meier survival analysis. Analyses were censored for death or final visit. Between group comparisons were performed using the log-rank test. Renal and patient survival was stratified by sex and Berden histological class. Cox regression analysis was used to calculate the hazard ratio (HR) of ESKD in males versus females, with adjustment for age, ANCA serology, creatinine at diagnosis and Berden histological class. P < 0.05 was considered statistically significant. All statistical analyses were performed using RStudio (Version 1.2.5001). The following R packages were used: *dplyr, forcats, tableone, ggplot2, survival* and *survminer.*

## Results

### Participant characteristics

332 patients met the inclusion criteria (Table 1), comprising 256 (77.1%) and 76 (22.9%) patients from the Irish and British cohorts, respectively. The mean age was 62 years (SD 15) and 194 (58.4%) were male, mainly Caucasian (92.2%). 217 (65.4%) were diagnosed with MPA, 92 (27.7%) GPA, 7 (2.1%) EGPA and 16 (4.8%) were unclassified AAV. 199 (60.1%) displayed MPO-ANCA positivity. Baseline creatinine was 278 µmol/L (IQR 158–450). Median follow-up time was 40.2 months (IQR 17.3–69.2). During this time, 73 (22%) reached ESKD and 47 (14.2%) died.

**Table 1:** Baseline characteristics in the total cohort and stratified by sex.

	All	Female	Male	*p*
N*	332	138 (41.6)	194 (58.4)	
Origin = RKD /RFH*	256/76 (77.1/22.9)	99/39 (71.7/28.3)	157/37 (80.9/19.1)	0.067
Age at diagnosis**	62.5 (14.7)	65.0 (14.8)	60.7 (14.4)	0.009
Ethnicity*				0.729
White	306 (92.2)	125 (90.6)	181 (93.3)	
Asian	12 (3.6)	6 (4.3)	6 ( 3.1)	
Black	6 (1.8)	4 (2.9)	2 ( 1.0)	
Mixed	3 (0.9)	1 (0.7)	2 ( 1.0)	
Other	5 (1.5)	2 (1.4)	3 ( 1.5)	
AAV phenotype*				0.18
MPA	217 (65.4)	96 (69.6)	121 (62.4)	
GPA	92 (27.7)	31 (22.5)	61 (31.4)	
EGPA	7 (2.1)	2 (1.4)	5 (2.6)	
ANCA vasculitis unclassified	16 (4.8)	9 (6.5)	7 (3.6)	
MPO-ANCA specificity*	199 (60.1)	92 (66.7)	107 (55.4)	0.052
Baseline creatinine (µmol/L)***	278 [158.0, 450.0]	263 [140.5, 412.0]	288 [165.5, 504.5]	0.065
Baseline eGFR (mL/min/1.73 m^2^)***		16 [9, 34]	19 [10, 38]	
Weight (kg)**	77.1 (22.9)	69.3 (16.2)	82.5 (25.2)	< 0.001
Berden histological class*				0.415
Crescentic	97 (31.7)	42 (33.1)	55 (30.7)	
Focal	90 (29.4)	37 (29.1)	53 (29.6)	
Mixed	79 (25.8)	36 (28.3)	43 (24.0)	
Sclerotic	40 (13.1)	12 ( 9.4)	28 (15.6)	
Follow up (months)***	40.2 [17.3, 69.2]	36.0 [15.5, 57.8]	41.5 [19.4, 75.7]	0.091
End-stage kidney disease*	73 (22.0)	24 (17.4)	49 (25.3)	0.116
Death*	47 (14.2)	21 (15.2)	26 (13.4)	0.758

Missing data: baseline creatinine for females (n = 20), males (n = 27), weight for females (n = 20), males (n = 23).

Rare Kidney Disease Registry (RKD), Ireland. Royal Free Hospital (RFH), London, U.K. Microscopic polyangiitis (MPA), Granulomatosis with polyangiitis (GPA), Eosinophilic granulomatosis with polyangiitis (EGPA), Estimated Glomerular Filtration Rate (CKD-EPI, eGFR) was calculated from baseline creatinine.

*Frequency (%).

**Mean (standard deviation).

***Median (inter-quartile range).

Table 1 displays baseline characteristics stratified by sex. Females were older (mean age 65 years in females versus 61 years in males, *p* 0.009) with a trend towards more MPO-ANCA positivity compared to males (66.7% versus 55.4%, *p* 0.052). Males had slightly better renal function at baseline (eGFR 19 (10–38) mL/min/1.73 m^2^ in males versus 16 (9–34) mL/min/1.73 m^2^ in females), but this was not statistically significant. Females were lighter (female mean weight 69.3 kg versus 82.5 kg in males, *p* < 0.001). There were no other significant differences in baseline characteristics between males and females.

### Renal survival

**Figure 1:**
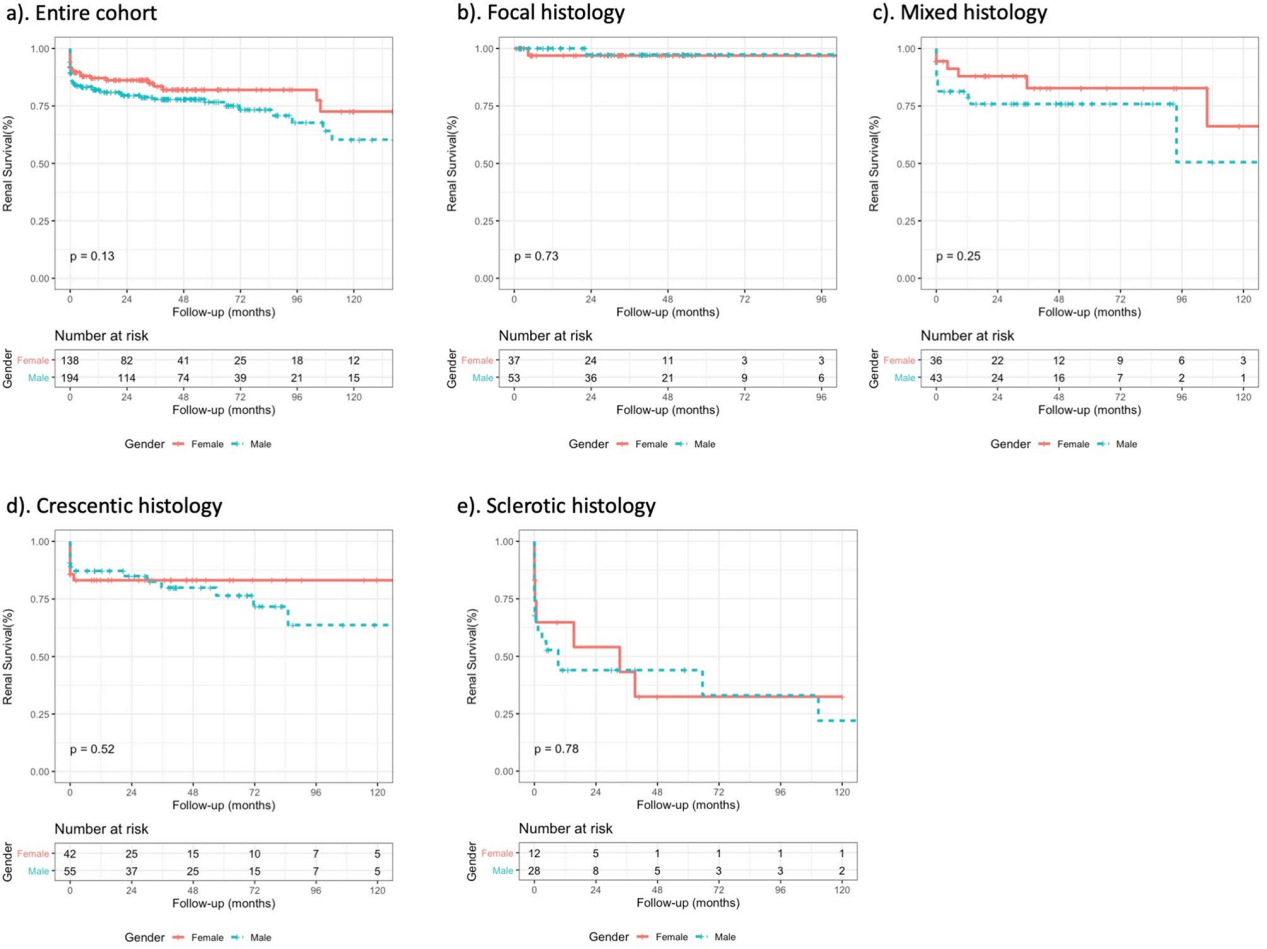
Kaplan–Meier plots demonstrating renal survival in males versus females in (**a**) the entire cohort, and in those with (**b**) focal histology, (**c**) mixed histology, (**d**) crescentic histology and (**e**) sclerotic histology.

Overall, renal survival was 84% (95% confidence interval (CI) 80–88) at 1 year and 79% (95% CI 74–84) at 5 years. We observed an excess of ESKD in males in the first six months after diagnosis, but the probability of ESKD was not significantly higher in males over the full period of observation (Fig. 1a). There was also no significant difference in renal survival between males and females when stratified by Berden histological class (Table 2 and Fig. 1b–e). The overall 1- and 5-year renal survival was 82.2% (95% CI 76.9–87.8) and 76.7% (95% CI 70.4–83.5) in males and 87.1% (95% CI 81.6–93.1) and 82.0% (95% CI 75.0–89.7) in females, respectively. The hazard ratio (HR) for ESKD in males versus females, after adjustment for age, ANCA serology, baseline creatinine and histological class was 1.07 (95% CI 0.59–1.93, *p* 0.82).

**Table 2:** Renal survival at 1- and 5-years follow-up, stratified by gender and Berden classification.

Characteristic	*N*		ESKD		1-year		5-year		*p* value
	Males	Females	Males	Females	Males (%, 95% CI)	Females (%, 95% CI)	Males (%, 95% CI)	Females (%, 95% CI)	
All	194	138	49	24	82.2 (76.9–87.8)	87.1 (81.6–93.1)	76.7 (70.4–83.5)	82.0 (75.0–89.7)	0.13
Focal	53	37	2	2	100 (1.0–1.0)	96.9 (91.0–1.0)	97.4 (92.4–1.0)	96.9 (91.0–1.0)	0.73
Mixed	43	36	11	7	81.4 (70.6–93.9)	87.9 (77.5–99.8)	75.9 (63.8–90.2)	82.8 (69.6–98.5)	0.25
Crescentic	55	42	13	7	87.2 (78.7–96.5)	83.1 (72.4–95.4)	76.4 (64.7–90.3)	83.1 (72.4–95.4)	0.52
Sclerotic	28	12	17	7	44.4 (28.4–68.0)	64.8 (421–99.8)	44.4 (28.4–68.0)	32.4 (13.1–80.4)	0.78
Missing*	15	11							

*Berden histological class missing in n = 26.

### Patient and renal survival

**Figure 2:**
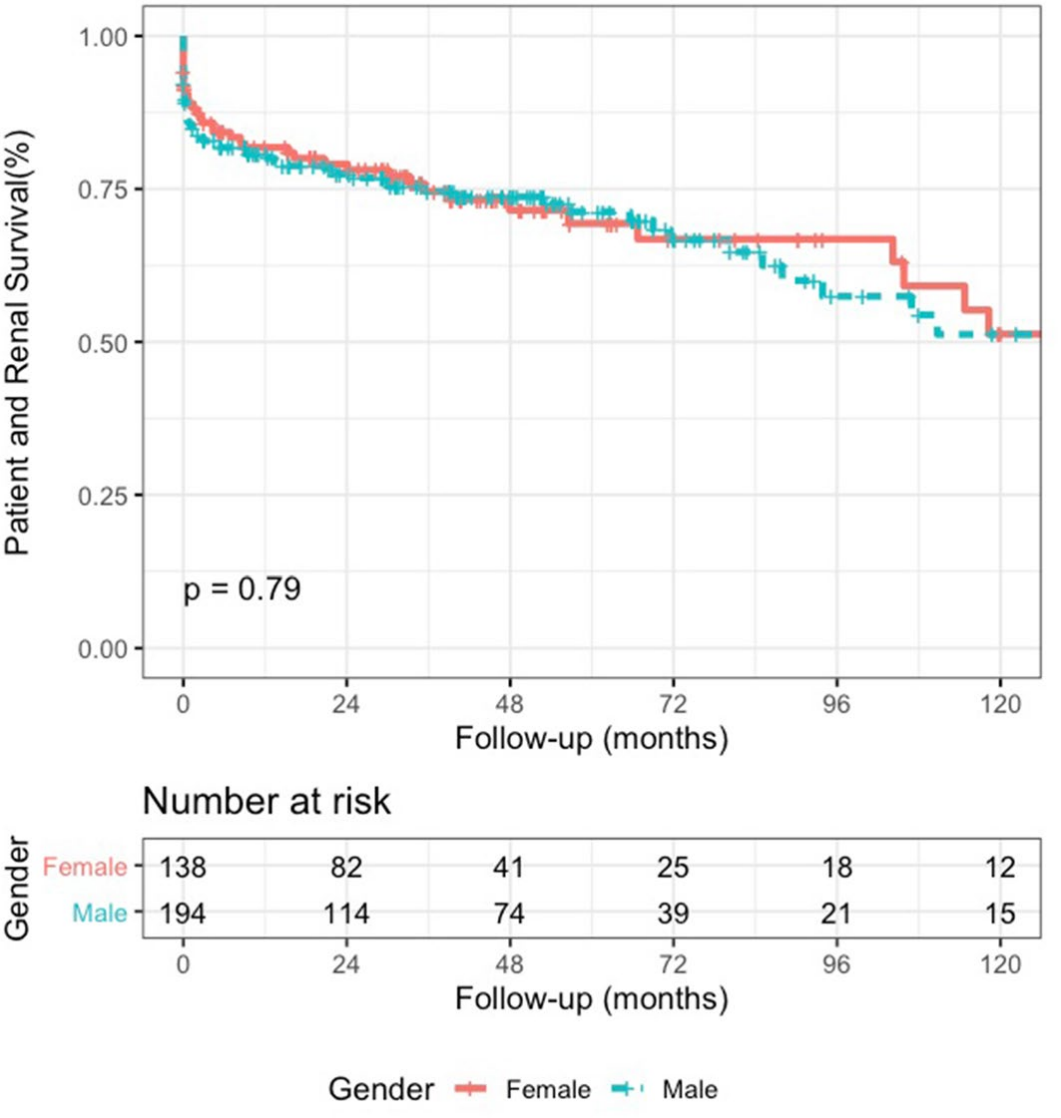
Kaplan–Meier plot demonstrating patient and renal survival in males versus females.

There was no difference in the composite outcome of patient and renal survival between sexes (Fig. 2), or when stratified by histological class (Table 3). Cox regression analysis showed that the HR for ESKD/death in males versus females, after adjustment for age, ANCA serology, baseline creatinine and histological class was 1.14 (95% CI 0.70–1.88, *p* 0.60).

**Table 3:** Patient and renal survival at 1- and 5-years follow-up, stratified by gender and Berden classification.

Characteristic	*N*		ESKD/death		1-year		5-year		*p* value
	Males	Females	Males	Females	Males (%, 95% CI)	Females (%, 95% CI)	Males (%, 95% CI)	Females (%, 95% CI)	
All	194	138	60	40	80.6 (75.2–86.4)	81.8 (75.4–88.7)	71.3 (64.5–78.8)	69.4 (60.6–79.4)	0.79
Focal	53	37	7	5	98.1 (94.4–100)	88.3 (78.1–99.8)	87.4 (77.4–98.6)	88.3 (78.1–99.8)	0.83
Mixed	43	36	12	13	81.4 (70.6–93.9)	85.0 (73.7–98.1)	69.5 (54.5–88.7)	66.6 (50.1–88.6)	0.96
Crescentic	55	42	16	12	85.3 (76.3–95.2)	75.7 (63.6–90.0)	74.8 (63.0–88.8)	72.8 (60.2–88.0)	0.93
Sclerotic	28	12	19	8	40.8 (25.8–64.7)	64.8 (42.1–99.8)	35.7 (21.1–60.6)	16.2 (3.1–85.0)	0.79
Missing*	15	11							

*Berden histological class missing in n = 26.

### Induction treatment stratified by sex

To test whether any observed effects of sex on survival may be influenced by standardised (non-weight based) dosing of immunosuppression, we analysed the weight adjusted doses delivered in those in whom complete data on individual induction immunosuppression was available (n = 189). The median cumulative dose per kilogram for intravenous cyclophosphamide, oral cyclophosphamide and rituximab is shown Table 4. There was no significant difference between males and females for any agent.

**Table 4:** Induction immunosuppression dose per kilogram, stratified by gender.

Drug (mg)	Median (IQR) (mg/kg)			
	N	Female	Male	*p* value
Cumulative IV cyclophosphamide dose/kg	81	58.15 (53.38, 73.89)	64.23 (51.52, 79.48)	0.45
Cumulative PO cyclophosphamide dose/kg	49	86.70 (68.15, 146.92)	126.11 (74.07, 201.53)	0.35
Cumulative rituximab dose/kg	59	30.77 (23.53, 38.46)	28.85 (21.17, 32.60)	0.11
Starting prednisolone dose/kg	200	0.77 (0.64, 0.76)	0.71 (0.57, 0.70)	0.05

Milligram (mg), kilogram (kg), intravenous (IV), oral (PO).

## Discussion

In our international, multi-centre study, we did not find a significant sex-specific difference in ANCA-GN outcomes. This contrasts with the increased risk of progression to ESKD for Norwegian males with ANCA-GN, reported by Bjørneklett et al.^14^. Our findings were similar when combining ESKD and death as a composite outcome: there was no difference between males and females.

Norwegian males with ANCA-GN, reported by Bjørneklett et al.^14^, were found to have a 2.44-fold increased risk of ESKD (HR 2.44, 95% CI 1.56–3.82, p < 0.001), after adjustment for age, ANCA serology, eGFR, and histological classification. This is consistent with prior observations in all-cause CKD where kidney function declines more rapidly and the risk of ESKD is higher in men^17,18^. Data from the Norwegian study was collected between 1991 and 2012 when the standard of care was oral cyclophosphamide. This differs from our study which commenced in 2012, after the publication of the CYCLOPS trial^19^, after which intravenous cyclophosphamide gained popularity. It has been postulated that women are more adherent to daily oral medication regimes, which may play a role in the difference observed by Bjørneklett et al.^14^, but not in the later pulsed intravenous regimes which predominate in our study. Importantly, like the previous study^14^, baseline kidney function in males was slightly better than in females, so this factor should not bias in favour of a better renal outcome in females, and baseline kidney function was included as a confounder in the multivariate model. Overall, our cohort had more advanced kidney disease at presentation: median baseline estimated Glomerular Filtration Rate (CKD-EPI, eGFR) 17.5 mL/min/1.73 m^2^, versus 34 mL/min/1.73 m^2^ in the Norwegian study. Despite this, and the higher proportion of MPO-ANCA positivity in our cohort (which in itself a risk factor for ESKD^20^), a similar proportion of patients in both cohorts progressed to ESKD, in keeping with that observed in other recent work^3^. This observation probably reflects the improvement in overall AAV care over the last few decades^3^, which may have eliminated the sex-specific discrepancy in renal outcome seen prior to 2012.

In contrast to the literature^14,21^, we identified that MPO-ANCA is more prevalent than PR3-ANCA in males. We also identified a higher proportion of MPO-ANCA positivity overall (60.1%) compared to the Norwegian study (46.0%), which is in keeping with the known latitudinal gradient of PR3:MPO ANCA specificity^21^. This highlights the heterogenous nature of AAV across different geographic regions, despite using identical inclusion criteria. This geographical diversity, potentially underpinned by genetics^22^, may account for inconsistencies in renal outcome across different cohorts.

We repeated the Kaplan–Meier analysis stratified for Berden histological class. In keeping with the original Berden description and the recent re-validation by van Daalen et al.^10^, we demonstrated that the focal subclass carries the best renal prognosis, while the sclerotic class performs the worst. The crescentic and mixed classes displayed similar prognosis. We did not observe an interaction between histological class and gender on ESKD risk. Notably, the large sex-specific difference observed by Bjørneklett et al.^14^ in patients with the crescentic subclass was not replicated in our cohort.

Our aim was to build on the findings of Bjørneklett et al.^14^ by using the very granular treatment data available to us, to explore whether their findings were due to sex-dependent variation in cumulative immunosuppression dosing, relative to body mass. We hypothesised that females received a higher effective induction immunosuppression dose due to their lower average body mass when fixed dosing regimens are administered. However, we found no difference between sexes in the cumulative dose normalised to body weight of any of the induction agents. It is possible we were underpowered to detect a difference as complete treatment data was missing in 43% of the cohort, but our evidence suggests sex-dependent variation in treatment intensity is not a factor in determining renal outcome.

To our knowledge, our study is the second of its kind to investigate sex-specific differences in renal outcomes in patients with AAV glomerulonephritis. Our large international multi-centre cohort study benefits from detailed longitudinal data including comprehensive individual treatment records. However, due to the observational nature of our data, there is a possibility that treatment data is incomplete, resulting in inaccurate conclusions. It also remains possible that our study is under-powered to detect small effects, although, if present, these would be unlikely to be clinically significant. To mitigate this we restricted this sub-analysis to include patients with a realistic cumulative dose (> 3 doses of IV cyclophosphamide or > 1 rituximab dose in the first 6 months of treatment). Study endpoints were obtained retrospectively from hospital records. Therefore, there is a possibility that death records are incomplete if the event occurred outside of the hospital. However, given that AAV patients are generally followed up in hospital settings, this is unlikely. In addition, sustained chronic kidney disease stage 5 is increasingly recognised as part of the ESKD definition, but was not included in our study (in line with the Norwegian study). We chose to use identical inclusion criteria and definitions to Bjørneklett et al.^14^ to enable accurate validation. Similarly, only biopsy proven cases were included, which results in selection bias towards more severe cases. It is possible that a significant difference in renal outcome exists between sexes when all patients with renal involvement are included*.*

In summary, we did not identify any clear sex-specific difference in renal outcome when stratified by Berden histological class. Furthermore, we found no evidence to support that any difference is due to sex-dependent variation in cumulative immunosuppression dosing. On the contrary, we found that weight-based dosing regimens are now standard practice in Ireland, and treatment intensity does not vary by sex. There is a potential to further explore gender differences in well characterized registries with granular longitudinal follow-up, and to utilize the expanding interoperable nature of our European registries through initiatives such as FAIRVASC^23^. When designing future multi-centre collaborative studies, we should consider the biopsychosocial influences of sex variance in AAV, in the context of outcomes and treatment- response and toxicity.

## Data Availability

The complete raw data underlying this article cannot be shared publicly due to the potentially identifiable nature of the data, due to the rarity of ANCA-associated vasculitis and the fact that the follow-up centres are published in this paper. Anonymised data will be shared on reasonable request to the corresponding author.

## References

[1] Tan JA (2017). Mortality in ANCA-associated vasculitis: A meta-analysis of observational studies. Ann. Rheum. Dis..

[2] Moiseev S, Novikov P, Jayne D, Mukhin N (2017). End-stage renal disease in ANCA-associated vasculitis. Nephrol. Dial. Transplant..

[3] Trejo MACW (2019). Renal relapse in antineutrophil cytoplasmic autoantibody-associated vasculitis: Unpredictable, but predictive of renal outcome. Rheumatology (U.K.).

[4] Rhee RL (2016). Trends in long-term outcomes among patients with antineutrophil cytoplasmic antibody-associated vasculitis with renal disease. Arthritis Rheumatol..

[5] Bjørneklett R, Sriskandarajah S, Bostad L (2016). Prognostic value of histologic classification of ANCA-associated glomerulonephritis. Clin. J. Am. Soc. Nephrol..

[6] Hilhorst M (2013). Estimating renal survival using the ANCA-associated GN classification. J. Am. Soc. Nephrol..

[7] Berden AE (2010). Histopathologic classification of ANCA-associated glomerulonephritis. J. Am. Soc. Nephrol..

[8] Chang DY (2011). Re-evaluation of the histopathologic classification of ANCA-associated glomerulonephritis: A study of 121 patients in a single center. Nephrol. Dial. Transplant..

[9] Tanna A (2014). Long-term outcome of anti-neutrophil cytoplasm antibody-associated glomerulonephritis: Evaluation of the international histological classification and other prognostic factors. Nephrol. Dial. Transplant..

[10] van Daalen EE (2020). Developments in the histopathological classification of ANCA-associated glomerulonephritis. Clin. J. Am. Soc. Nephrol..

[11] Watts RA (2015). Classification, epidemiology and clinical subgrouping of antineutrophil cytoplasmic antibody (ANCA)-associated vasculitis. Nephrol. Dial. Transplant..

[12] Jennette JC, Nachman PH (2017). ANCA glomerulonephritis and vasculitis. Clin. J. Am. Soc. Nephrol..

[13] Jennette JC (2003). Rapidly progressive crescentic glomerulonephritis. Kidney Int..

[14] Bjørneklett R, Solbakken V, Bostad L, Fismen AS (2018). Exploring sex-specific differences in the presentation and outcomes of ANCA-associated vasculitis: A nationwide registry-based cohort study. Int. Urol. Nephrol..

[15] https://www.tcd.ie/medicine/thkc/research/rare.php, https://www.tcd.ie/medicine/thkc/research/rare.php.

[16] Watts R (2007). Development and validation of a consensus methodology for the classification of the ANCA-associated vasculitides and polyarteritis nodosa for epidemiological studies. Ann. Rheum. Dis..

[17] Carrero JJ, Hecking M, Chesnaye NC, Jager KJ (2018). Sex and gender disparities in the epidemiology and outcomes of chronic kidney disease. Nat. Rev. Nephrol..

[18] Minutolo R (2020). Sex Differences in the progression of CKD among older patients: Pooled analysis of 4 cohort studies. Am. J. Kidney Dis..

[19] de Groot K (2009). Pulse versus daily oral cyclophosphamide for induction of remission in antineutrophil cytoplasmic antibody—associated vasculitis. Ann. Intern. Med..

[20] Flossmann O (2011). Long-term patient survival in ANCA-associated vasculitis. Ann. Rheum. Dis..

[21] Weiner M (2019). Proteinase-3 and myeloperoxidase serotype in relation to demographic factors and geographic distribution in anti-neutrophil cytoplasmic antibody-associated glomerulonephritis. Nephrol. Dial. Transplant..

[22] Watts RA, MacGregor AJ, Mackie SL (2014). HLA allele variation as a potential explanation for the geographical distribution of granulomatosis with polyangiitis. Rheumatology.

[23] https://fairvasc.eu/the-project/, https://fairvasc.eu/the-project/.

